# Effectiveness of influenza vaccines in children aged 6 to 59 months: a test-negative case–control study at primary care and hospital level, Spain 2023/24

**DOI:** 10.2807/1560-7917.ES.2024.29.40.2400618

**Published:** 2024-10-03

**Authors:** Gloria Pérez-Gimeno, Clara Mazagatos, Nicola Lorusso, Luca Basile, Isabel Martínez-Pino, Francisca Corpas Burgos, Noa Batalla Rebolla, Mercedes B Rumayor Zarzuelo, Blanca Andreu Ivorra, Jaume Giménez Duran, Daniel Castrillejo, Inés Guiu Cañete, Marta Huerta Huerta, Marta García Becerril, Violeta Ramos Marín, Inmaculada Casas, Francisco Pozo, Susana Monge, the SiVIRA group, Members of the SiVIRA sentinel surveillance study group, Alba Moya Garcés, Esteban Pérez, Luis García Comas, Paloma Botella Rocamora, Santiago Vicente Iglesias, Ana Ordax Díez, Lourdes Duro Gómez, Mª Olga Hidalgo Pardo, Sergio Román-Soto, Ana D. Cebollada Gracia, Ana Fernandez Ibáñez, Manuel Galán Cuesta, Ninoska López Berrios, Carlota Ruiz Porras, Irene Pedrosa Corral, Marta Pérez Abeledo, Ana S. Lameiras Azevedo, Sofía García Senso, Virginia Álvarez Río, Mª Ángel Valcárcel de Laiglesia, Jorge Reina Prieto, Francisco J. Vega-Olías, Miriam García Vázquez, Pilar Alonso Vigil, Luis J. Viloria Raymundo, Paulina V. Medel Jaime, Marcos Lozano, Lorena Vega-Piris, Silvia Galindo-Carretero

**Affiliations:** 1 Department of Communicable Diseases, National Centre of Epidemiology, Institute of Health Carlos III, Madrid, Spain; 2 Consortium for Biomedical Research in Epidemiology and Public Health (CIBERESP), Madrid, Spain; 3 Servicio de Vigilancia y Salud Laboral. Dirección General de Salud Pública y Ordenación Farmacéutica, Consejería de Salud y Consumo, Andalucía, Spain; 4 Agència de Salut Pública de Catalunya, Generalitat de Catalunya, Barcelona, Spain; 5 Public Health General Directorate, Castilla y León Regional Ministry of Health, Valladolid, Spain; 6 Subdirecció General d’Epidemiologia i Vigilància de la Salut, Direcció General de Salut Pública, Generalitat Valenciana, Valencia, Spain; 7 Subdirección de Epidemiología de la Dirección General de Salud Pública, Servicio Extremeño de Salud, Merida, Spain; 8 Área de Enfermedades Transmisibles, Subdirección General de Vigilancia en Salud Pública. Dirección General de Salud Pública de la Comunidad de Madrid, Madrid, Spain; 9 Servicio de Epidemiología (Sección Vigilancia Epidemiológica), Consejería de Salud- Región de Murcia, Murcia, Spain; 10 Servicio de Epidemiologia, Consellería de Salut, Gobierno de las Islas Baleares, DGSP Baleares (IDISBA), Palma, Spain; 11 Servicio de Vigilancia Epidemiológica, Dirección General de Salud Pública, Melilla, Spain; 12 Sección de Vigilancia Epidemiológica de la Dirección General de Salud Pública, Aragón, Spain; 13 Dirección General de Salud Pública y Atención a la Salud Mental, Consejería de Sanidad, Principado de Asturias, Spain; 14 Dirección General de Salud Pública de Cantabria, Santander, Spain; 15 Servicio de Epidemiología de la Consejería de Sanidad y Servicios Sociales de Ceuta, Ceuta, Spain; 16 National Centre for Microbiology, Institute of Health Carlos III, Madrid, Spain; 17 Consortium for Biomedical Research on Infectious Diseases (CIBERINFEC), Madrid, Spain; 18 The members of the network are listed under Collaborators

**Keywords:** influenza, vaccine effectiveness, children, test-negative design

## Abstract

During 2023/24, all children aged 6 to 59 months were targeted for seasonal influenza vaccination in Spain nationally. Using a test-negative case–control design with sentinel surveillance data, we estimated adjusted influenza vaccine effectiveness (IVE) against any influenza type to be 70% (95% confidence interval (CI): 51 to 81%) for primary care patients with acute respiratory illness (ARI) and 77% (95% CI: 21 to 93%) for hospitalised patients with severe ARI. In primary care, where most subtyped viruses (61%; 145/237) were A(H1N1), adjusted IVE was 77% (95% CI: 56 to 88%) against A(H1N1)pdm09.

It is estimated that globally, 109 million influenza virus infections occur annually in 0 to 59-month-old children [1]; in this age group, infection can lead to severe disease. In Spain, children under 5 years of age have the second highest rate of hospitalisation admission due to influenza, only below that of people 65 years or older [2]. Moreover, it has been reported that children play key roles in the community circulation of the virus and in the amplification of influenza epidemics [3]. Influenza vaccination in Spain was recommended in the 2023/24 season, for the first time at national level, to all children aged 6 to 59 months [4].

For children aged 6 to 59 months during the 2023/24 season, we estimated influenza vaccine effectiveness (IVE) against acute respiratory infections (ARI) in those attending primary care (PC), or against severe ARI (SARI) in those hospitalised, overall and by influenza virus type, subtype, and clade.

## Study setting

In Spain, the 2023/24 season was characterised by the circulation of influenza A. In both PC and hospitals, the A(H1N1)pdm09 subtype was predominant, followed by A(H3N2), while influenza B was scarcely detected. The children vaccination campaign started on week 39 2023 with one dose of tetravalent vaccines, inactivated (Influvac Tetra, Vaxigrip Tetra, Flucelvax Tetra, and Fluarix Tetra) or intranasal live attenuated egg-based (Fluenz Tetra) [4], which was provided free of charge at PC. The national vaccination coverage was 31.16% [5].

## Data sources, study design and eligibility criteria

The Surveillance System of Acute Respiratory Infections in Spain (SiVIRA) monitors ARI at PC level and SARI at hospital level. ARI is defined as sudden onset of at least one symptom among cough, sore throat, shortness of breath and/or rhinorrhoea (with or without fever ≥ 38°C) and a clinician’s judgment that the illness is due to an infection. SARI is an ARI that required hospitalisation for ≥ 24 hours [6,7]. Physicians systematically select for swabbing (and reverse-transcription (RT)-PCR virological testing) the first two to five ARI patients each week at PC and all patients hospitalised with SARI on pre-defined weekdays (Tuesdays and/or Wednesdays). Clinical, epidemiological, vaccination and virological data are also collected. Specimens testing positive for influenza are recommended to be typed and subtyped. National Influenza Centres and the National Centre of Microbiology sequenced the influenza viruses.

**Figure 1:**
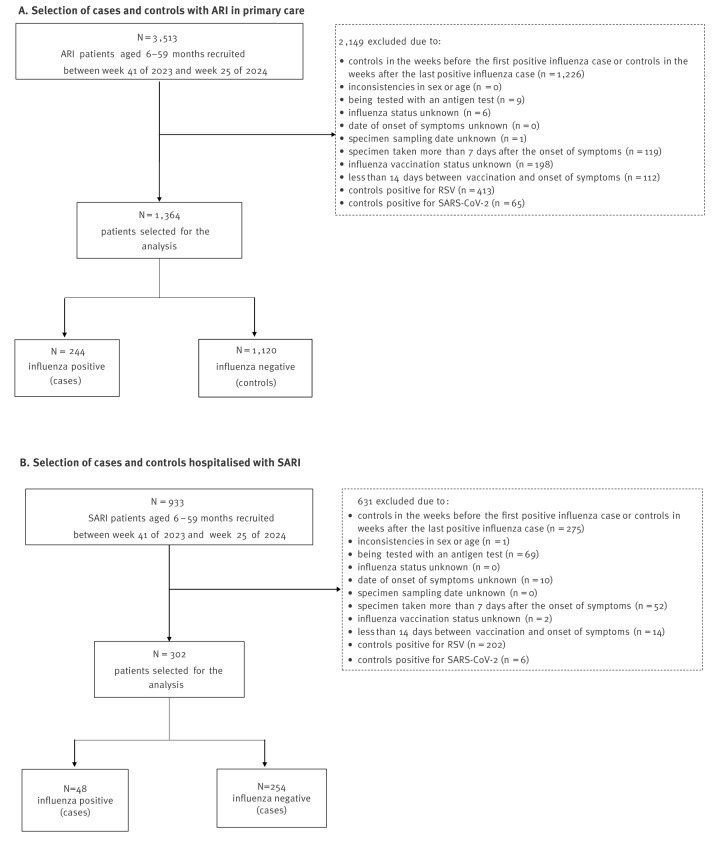
Flowchart of selection of paediatric cases and controls^a^ eligible for the sentinel surveillance, to estimate vaccine effectiveness against influenza virus-caused A) acute respiratory infections (ARI) in primary care and B) severe ARI (SARI) in inpatients, Spain, September 2023–June 2024 (n = 27 hospitals in 12 regions)

ARI: acute respiratory symptoms; RSV: respiratory syncytial virus; SARI: severe ARI; SARS-CoV-2: severe acute respiratory coronavirus 2.

^a^ Children aged 6–59 months.

We conducted a test-negative case–control study, using SiVIRA data from 12 of 19 regions in Spain and 27 hospitals. We included all children aged 6–59 months who attended PC with ARI or were hospitalised with SARI between week 41 2023 (14 days after onset of vaccination campaign) and week 25 2024, had a specimen collected within 7 days of symptoms onset, and had a valid RT-PCR result for influenza. Full selection criteria are outlined in Figure 1. Participants were classified as cases if they tested positive and as controls if they tested negative. Influenza vaccination data were collected from regional vaccination records, and vaccinations administered at least 14 days before symptoms onset were considered.

## Patients’ characteristics

**Figure 2:**
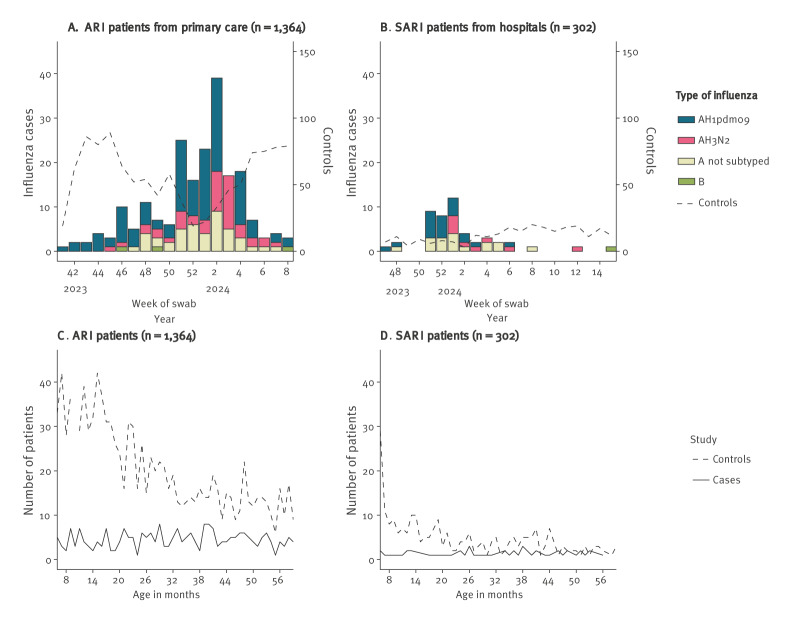
Number of paediatric cases and controls^a^ selected in the study by week and type/subtype of influenza among A) patients from primary care with acute respiratory infections (ARI), and B) hospitalised patients with severe ARI (SARI), and by age in months among C) ARI and D) SARI, Spain, September 2023–June 2024 (n = 27 hospitals in 12 regions)

ARI: acute respiratory symptoms; SARI: severe ARI.

^a^ Children aged 6–59 months.

We included 1,364 ARI patients in PC (244 cases), and 302 SARI patients in hospitals (48 cases). In both settings, controls were younger (p < 0.001), and in the PC setting, controls had higher influenza vaccination coverage (p = 0.001). In both settings, influenza B exhibited low circulation (Figure 2, Table 1). For influenza A viruses that were subtyped, A(H1N1)pdm09 dominated (61.2% and 43.8% in PC and hospitals, respectively), followed by A(H3N2) (19% and 18.8% respectively).

**Table 1:** Characteristics of acute respiratory infection and severe acute respiratory infection paediatric influenza cases and controls aged 6 to 59 months selected into the study, Spain, September 2023–June 2024 (n = 27 hospitals in 12 regions)

Characteristic	ARI patients (n = 1,364)								SARI patients (n = 302)						
	Cases (n = 244)			Controls (n = 1,120)				p^a^	Cases (n = 48)			Controls (n = 254)			p^a^
**Descriptive statistic**	**Median**		**IQR**	**Median**			**IQR**	**p**	**Median**		**IQR**	**Median**		**IQR**	**p**
**Age in months**	33		21–45	23			13–38	0.000	34.5		18.5–41	19		10–38	0.000
**Descriptive statistic**	**n**	**Denom.^b^**	**%**	**n**		**Denom.^b^**	**%**	**p**	**n**	**Denom.^b^**	**%**	**n**	**Denom.^b^**	**%**	**p**
**Sex^c^**															
**Female**	107	244	43.9	523		1,120	46.7	0.419	20	48	41.7	113	254	44.5	0.651
**Male**	137	244	56.1	597		1,120	53.3		28	48	58.3	141	254	55.5	
**Presence of chronic condition**															
**One or more**	8	171	4.7		62	839	7.4	0.094	11	42	26.2	59	190	31.1	0.556
**Hypertension**	0	168	0		0	840	0	NA	0	47	0	1	237	0.4	0.696
**Chronic cardiovascular disease**	0	168	0		4	842	0.5	0.387	2	48	4.2	10	239	4.2	0.668
**Chronic respiratory disease**	6	182	3.3		56	900	6.2	0.088	6	48	12.5	35	235	14.9	0.510
**Diabetes**	0	168	0		0	842	0	NA	0	43	0	5	221	2.3	0.383
**Chronic liver disease**	0	168	0		1	843	0.1	0.666	0	47	0	0	230	0	NA
**Chronic kidney disease**	0	168	0		0	842	0	0	1	43	2.3	3	221	1.4	0.501
**Immunodeficiencies**	1	168	0.6		2	842	0.2	0.401	0	44	0	3	185	1.6	0.455
**Other chronic disease**	1	168	0.6		0	836	0	0.000	3	45	6.7	27	201	13.4	0.426
**Seasonal influenza vaccination**															
**Yes**	59	244	24.2		394	1,120	35.2	0.001	8	48	16.7	92	254	36.2	0.081
**Influenza type (subtype)**															
**A(H1N1)**	145	237	61.2		NA	NA	NA	NA	21	48	43.8	NA	NA	NA	NA
**A(H3N2)**	45	237	19.0		NA	NA	NA	NA	9	48	18.8	NA	NA	NA	NA
**A (unsubtyped)**	44	237	18.6		NA	NA	NA	NA	17	48	35.4	NA	NA	NA	NA
**B**	3	237	1.3		NA	NA	NA	NA	1	48	2.1	NA	NA	NA	NA

ARI: acute respiratory infection; denom.: denominator; IQR: interquartile range; NA: not applicable; SARI: severe ARI.

^a^ Pearson’s chi-square test.

^b^ Denominators include the number of cases with available information on the characteristic in question.

^c^ Data on sex were collected as male, female and not reported.

## Vaccine effectiveness

The odds of being vaccinated was compared between influenza cases and controls through an odds ratio (OR) and its 95% confidence interval (95% CI) using logistic regression and a penalised logistic method (Firth’s method) when the number of cases vaccinated was less than 10 [8].

Estimates were adjusted for potential confounders including sex, age in months, epidemiological week, presence of chronic conditions, and region or hospital for ARI or SARI models, respectively; IVE = (1 − OR) × 100. IVE was estimated by type/subtype/clade when the sample size allowed, and only considering the weeks with positive cases for the respective type/subtype/clade.

Among ARI patients in PC, IVE against any influenza infection was 70% (95% CI: 51 to 81) (Table 2). IVE was 77% (95% CI: 56 to 88) against A(H1N1)pdm09, which circulated predominately (61%), and higher against clade 5a.2a at 96% (95% CI: 23 to 100), while no protection could be demonstrated against A(H3N2) or other clades, possibly related to the low number of cases and the extremely wide CIs. Among hospitalised SARI patients, the point estimate was 77% (95% CI: 21 to 93) against any influenza, and the estimated IVE by subtype had very low precision, with CIs including the null. Clade analyses could not be performed among hospitalised patients.

**Table 2:** Crude and adjusted influenza vaccine effectiveness with 95% CI against acute respiratory infections (ARI) in primary care and severe ARI (SARI) in hospitals for patients aged 6 to 59 months, overall, by type/subtype, and by clade of influenza virus, Spain, September 2023–June 2024 (n = 27 hospitals in 12 regions)

Setting and influenza type subtype or clade		Cases vaccinated/ total cases	Controls vaccinated/ total controls^a^	Crude IVE (%)	95% CI	Adjusted IVE (%)^b^	95% CI
**Primary care consultation with ARI**							
Main analysis	Any influenza	59/244	394/1,120	41	19 to 57	70	51 to 81
	A(H1N1)pdm09	30/145	394/1,120	52	27 to 68	77	56 to 88
	A(H3N2)	20/44	333/705	7	−72 to 49	18	−97 to 65
	B	0/3	371/784	NA	NA	NA	NA
Clade-specific analysis	5a.2a (H1N1)	3/22	221/814	58	−45 to 88	96	23 to 100
	5a.2a.1 (H1N1)	3/11	221/733	13	−231 to 77	49	−184 to 91
	2a.3a.1 (H3N2)	9/12	288/535	−157	−861 to 31	−116	−824 to 50
**Hospitalisation due to SARI**							
Any influenza		8/48	92/254	65	22 to 84	77	21 to 93
A(H1N1)pdm09		3/21	44/109	75	11 to 93	75	−68 to 96
A(H3N2)		4/9	67/169	−22	−370 to 68	−3	−563 to 84
B		0/1	4/12	NA	NA	NA	NA

ARI: acute respiratory infection; CI: confidence interval; IVE: influenza vaccine effectiveness; NA: not applicable as the number of cases vaccinated was insufficient to perform estimates; SARI: severe ARI.

^a^ Controls were excluded in weeks with no circulation of the specific influenza subtype or clade.

^b^ Logistic regression adjusted by sex, age in months (as restricted cubic spline), epidemiological week (as restricted cubic spline), region and presence of chronic conditions.

Two sensitivity analyses were conducted. First, including controls positive for severe acute respiratory syndrome coronavirus 2 (SARS-CoV-2) and/or respiratory syncytial virus (RSV) changed estimates: in PC, to 68% (95% CI: 49 to 79) against overall influenza, 76% (95% CI: 55 to 87) against A(H1N1)pdm09 and 2% (95% CI: −130 to 58) against A(H3N2), and in hospitals to 76% (95% CI: 26 to 92), 78% (95% CI: −25 to 96) and 19% (95% CI: −374 to 86), respectively. Second, categorising unknown vaccination status as unvaccinated only changed IVE estimate in hospitals to 86% (95% CI: 39 to 97) against overall influenza, 76% (95% CI: −65 to 96) against A(H1N1)pdm09 and −7% (95% CI: −609 to 84) against A(H3N2).

## Discussion

We found high IVE in children aged 6–59 months against influenza attended in PC, particularly against A(H1N1)pdm09 and clade 5a.2a, but we lacked sufficient sample size to provide accurate estimates of IVE against other subtypes and clades.

Despite the differences in the target age groups, our results are comparable with those of an interim 2023/24 investigation in children under 17 years, which found an overall similar IVE of 71% through the European PC multicentre study, and slightly higher IVE of 53% through the hospital multicentre study [9]. Early IVE estimates from Canada for A(H1N1) in children aged 6 months to 9 years both hospitalised and not hospitalised (74%), were also similar to ours [10]. The United States interim 2023/24 study, however, reported lower IVEs, ranging from 59% to 67% in outpatients below 17 years old in PC and ranging from 52% to 61% in hospitalised children aged 6 months to 17 years [11].

Our results indicated lower effectiveness against clade 5a.2a.1, although with very low precision and a 95% CI including the null. This is compatible with last season antigenic human studies, that had motivated World Health Organization (WHO) to change the 2023/24 vaccine clade recommendation from 5a.2a to 5a.2a.1 [12]. Skowronski et al. suggested that a mutation in the 2023/24 clade 5a.2a.1 vaccine high-growth reassortant, specifically the R142K(Ca2) reversion, may be responsible for reduced IVE [13]. This mutation affected only the high-growth reassortant IVR-238 used for egg-based vaccines, so comparing IVE between different vaccine brands against the same clade could provide more information. Unfortunately, we could not test this hypothesis.

Our study has other limitations inherent to observational studies and unmeasured confounders.

## Conclusion

In conclusion, our results along with previously available evidence supports the effectiveness of influenza vaccination in children aged 6–59 months to prevent both influenza infection and hospitalisation. Continued efforts are needed to increase coverage of influenza vaccination in this age group in future seasons.

## References

[1] Wang X Li Y O’Brien KL Madhi SA Widdowson MA Byass P Respiratory Virus Global Epidemiology Network . Global burden of respiratory infections associated with seasonal influenza in children under 5 years in 2018: a systematic review and modelling study. Lancet Glob Health. 2020;8(4):e497-510. 10.1016/S2214-109X(19)30545-5 32087815 PMC7083228

[2] Grupo de trabajo de Recomendaciones de Vacunación frente a gripe en población infantil de 6 a 59 meses de la Ponencia de Programa y Registro de Vacunaciones. Comisión de Salud Pública del Consejo Interterritorial del Sistema Nacional de Salud. [Working Group on Vaccination Recommendations for Influenza in Children Aged 6 to 59 Months from the Vaccination Program and Registry Committee]. Madrid: Ministerio de Sanidad, Oct 2022. Spanish. Available from: https://www.sanidad.gob.es/areas/promocionPrevencion/vacunaciones/programasDeVacunacion/docs/Recomendaciones_vacunacion_gripe_PoblacionInfantil.pdf

[3] Nayak J Hoy G Gordon A . Influenza in Children. Cold Spring Harb Perspect Med. 2021;11(1):a038430. 10.1101/cshperspect.a038430 31871228 PMC7778215

[4] Ponencia de Programa y Registro de Vacunaciones. Recomendaciones de vacunación frente a gripe y COVID-19 en la temporada 2023-2024 en España. Actualización. [Recommendations against influenza and COVID-19 in the 2023/24 season in Spain; update]. 2023 sep. Spanish. Available from: https://www.sanidad.gob.es/areas/promocionPrevencion/vacunaciones/programasDeVacunacion/docs/Recomendaciones_vacunacion_gripe.pdf

[5] Ministerio de Sanidad. Sistema de Información de Vacunaciones del Ministerio de Sanidad (SIVAMIN), Aplicación interactiva. [Ministry of Health. Vaccination Information System of the Ministry of Health (SIVAMIN), Interactive Application]. Spanish. [Accessed 01 Oct 2024]. Available from: https://pestadistico.inteligenciadegestion.sanidad.gob.es/publicoSNS/I/sivamin/sivamin

[6] Red Nacional de Vigilancia Epidemiológica (RENAVE), Centro Nacional de Epidemiología, Centro Nacional de Microbiología. Instituto de Salud Carlos III. Protocolo para la vigilancia centinela de Infección respiratoria aguda (IRAs) en Atención Primaria en España Temporada 2023-24. [Protocol for Sentinel Surveillance of Acute Respiratory Infections (ARI) in Primary Care in Spain for the 2023-24 Season]. 2023. Spanish. Available from: https://www.isciii.es/QueHacemos/Servicios/VigilanciaSaludPublicaRENAVE/EnfermedadesTransmisibles/Documents/GRIPE/Protocolos/Protocolo%20Vigilancia%20centinela%20de%20IRAs_2023-24_v.27092023.pdf

[7] Red Nacional de Vigilancia Epidemiológica (RENAVE), Centro Nacional de Epidemiología, Centro Nacional de Microbiología. Instituto de Salud Carlos III. Protocolo para la vigilancia centinela de infección respiratoria aguda grave (IRAG) en hospitales en España Temporada 2023-24. [Protocol for Sentinel Surveillance of Severe Acute Respiratory Infections (SARI) in Hospitals in Spain for the 2023-24 Season]. 2023. Spanish. Available from: https://www.isciii.es/QueHacemos/Servicios/VigilanciaSaludPublicaRENAVE/EnfermedadesTransmisibles/Documents/GRIPE/Protocolos/Protocolo_Vigilancia%20centinela%20de%20IRAG_2023-24_v.27092023.pdf</eref>

[8] Peduzzi P Concato J Kemper E Holford TR Feinstein AR . A simulation study of the number of events per variable in logistic regression analysis. J Clin Epidemiol. 1996;49(12):1373-9. 10.1016/S0895-4356(96)00236-3 8970487

[9] Maurel M Howard J Kissling E Pozo F Pérez-Gimeno G Buda S European IVE group . Interim 2023/24 influenza A vaccine effectiveness: VEBIS European primary care and hospital multicentre studies, September 2023 to January 2024. Euro Surveill. 2024;29(8):2400089. 10.2807/1560-7917.ES.2024.29.8.2400089 38390651 PMC10899813

[10] Smolarchuk C Ickert C Zelyas N Kwong JC Buchan SA . Early influenza vaccine effectiveness estimates using routinely collected data, Alberta, Canada, 2023/24 season. Euro Surveill. 2024;29(2):2300709. 10.2807/1560-7917.ES.2024.29.2.2300709 38214082 PMC10785209

[11] Frutos AM Price AM Harker E Reeves EL Ahmad HM Murugan V CDC Influenza Vaccine Effectiveness Collaborators . Interim Estimates of 2023-24 Seasonal Influenza Vaccine Effectiveness - United States. MMWR Morb Mortal Wkly Rep. 2024;73(8):168-74. 10.15585/mmwr.mm7308a3 38421935 PMC10907036

[12] World Health Organization (WHO). Recommended composition of influenza virus vaccines for use in the 2023-2024 northern hemisphere influenza season. Geneva: WHO; Feb 2023. Available from: https://www.who.int/publications/m/item/recommended-composition-of-influenza-virus-vaccines-for-use-in-the-2023-2024-northern-hemisphere-influenza-season

[13] Skowronski DM Zhan Y Kaweski SE Sabaiduc S Khalid A Olsha R 2023/24 mid-season influenza and Omicron XBB.1.5 vaccine effectiveness estimates from the Canadian Sentinel Practitioner Surveillance Network (SPSN). Euro Surveill. 2024;29(7):2400076. 10.2807/1560-7917.ES.2024.29.7.2400076 38362622 PMC10986657

